# Central Venous Catheter Intravascular Malpositioning: Causes, Prevention, Diagnosis, and Correction

**DOI:** 10.5811/westjem.2015.7.26248

**Published:** 2015-10-20

**Authors:** Carlos J. Roldan, Linda Paniagua

**Affiliations:** University of Texas Health Science Center, Department of Emergency Medicine, Houston, Texas

## Abstract

Despite the level of skill of the operator and the use of ultrasound guidance, central venous catheter (CVC) placement can result in CVC malpositioning, an unintended placement of the catheter tip in an inadequate vessel. CVC malpositioning is not a complication of central line insertion; however, undiagnosed CVC malpositioning can be associated with significant morbidity and mortality. The objectives of this review were to describe factors associated with intravascular malpositioning of CVCs inserted via the neck and chest and to offer ways of preventing, identifying, and correcting such malpositioning. A literature search of PubMed, Cochrane Library, and MD Consult was performed in June 2014. By searching for “Central line malposition” and then for “Central venous catheters intravascular malposition,” we found 178 articles written in English. Of those, we found that 39 were relevant to our objectives and included them in our review. According to those articles, intravascular CVC malpositioning is associated with the presence of congenital and acquired anatomical variants, catheter insertion in left thoracic venous system, inappropriate bevel orientation upon needle insertion, and patient’s body habitus variants. Although plain chest radiography is the standard imaging modality for confirming catheter tip location, signs and symptoms of CVC malpositioning even in presence of normal or inconclusive conventional radiography findings should prompt the use of additional diagnostic methods to confirm or rule out CVC malpositioning. With very few exceptions, the recommendation in cases of intravascular CVC malpositioning is to remove and relocate the catheter. Knowing the mechanisms of CVC malpositioning and how to prevent, identify, and correct CVC malpositioning could decrease harm to patients with this condition.

## INTRODUCTION

Central venous catheters (CVCs) are cannulation devices designed to access the central venous circulation and are inserted via wire guidance (i.e., via the Seldinger technique). In the emergent setting, CVCs are used to administer life-supporting fluids, potentially irritant drugs, blood products, and parenteral nutrition. In other settings, CVCs are used to provide access for hemodialysis, transvenous heart pacing, and monitoring of hemodynamics by measuring central filling pressure and cardiac output.^1^ CVC placement requires training and experience and is not without risk for patients, even when performed by skilled professionals.

The most common adverse events associated with neck and thorax CVC insertion have been extensively addressed in the literature and include infection (5% to 26%), hematoma (2% to 26%), and pneumothorax (up to 30%).^2^ Other complications of CVC placement include hemothorax, chylothorax, and extravasation of infusate, unrecognized arterial placement, cardiac tamponade, and mediastinal hemorrhage.^3^–^6^ A less commonly described yet important complication of CVC placement is malpositioning of the tip of the CVC in a vessel other than the superior vena cava (SVC). This event has been described in approximately 7% of cases of thoracic CVC placement in the literature^3^ and can lead to serious complications if not addressed. Placing the CVC tip in a vessel other than the SVC increases the risks of catheter wedging, erosion or perforation of vessel walls, local venous thrombosis, catheter dysfunction, and cranial retrograde injection, in which the infusate is directed to the head instead of the central circulation.^4^

The objectives of this review were to characterize the factors associated with neck and thorax CVC malpositioning and to offer ways of preventing, identifying, and correcting this error.

## LITERATURE SEARCH

We performed a literature search of PubMed, Cochrane Library, and MD Consult in June 2014. By searching for “Central line malposition” we found 188 articles in PubMed, one article in the Cochrane Library, and one in MD Consult. By searching for “Central venous catheters intravascular malposition” we found seven articles in PubMed, none in the Cochrane Library, and one article in MD consult. Of these, we reviewed 178 articles written in English. We first selected the articles published in the past 10 years whose content was directly relevant to the objective of our review and then included a few older relevant articles that the articles published in the past 10 years had cited. We thus included 39 articles in our review.

## MECHANISMS OF CVC MALPOSITIONING

While the mechanisms of CVC malpositioning are not well understood, it appears to be multifactorial. Some studies have shown that upon needle insertion, the bevel orientation facilitates the progression of the guide wire in the intended direction.^7^ For example, when one attempts an internal jugular vein catheterization, orienting the needle bevel medially facilitates guide wire passage into the SVC.^8^ With the same rationale, there have been small randomized controlled studies demonstrating an effect of bevel orientation in subclavian catheterizations, with a higher rate of correct placements when the bevel was oriented caudally.^8^ Similarly, orienting the bevel medially when attempting internal jugular vein insertion may maximize the success rate.

Some hypothesize that difficult body habitus (e.g., obesity or large breasts) can contribute to tip migration and increase the risk of malpositioning. When the external segment of a catheter is sutured in redundant tissue and the patient changes position from supine to upright, the mediastinal structures lengthen and the abdominal contents descend, causing relative cephalad pulling of the catheter tip with respect to the SVC and right atrium. Indeed, it has been radiographically demonstrated that the catheter tip can significantly move up cephalad, from mid-right atrium to low SVC, when the patient sits up; this migration was greater for CVC placed in the subclavian veins in females and in obese patients.^9^ Mild tip migration has also been described in association with breathing movements. A mean variation of 9mm of catheter tip movement was observed in expiration, but not in inspiration.^10^ These positional and breathing related variations can be of combined effects and be of more clinical significance when the catheter tip is placed too far from the right atrium or in patients with vascular anatomical variants.

**Figure 1:**
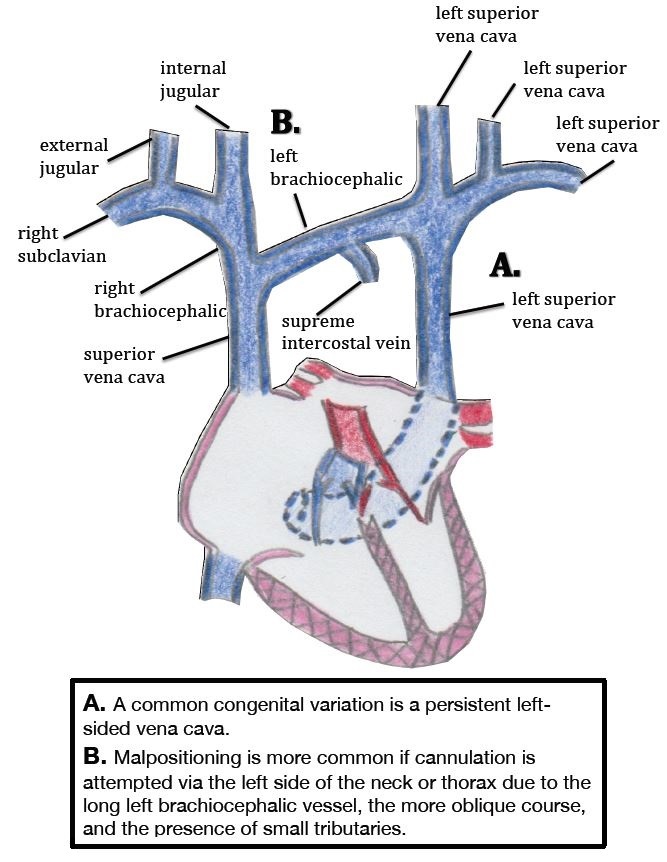
Common variants of clinical significance in the central venous anatomy, the congenital persistent left-sided superior vena cava.

**Figure 2:**
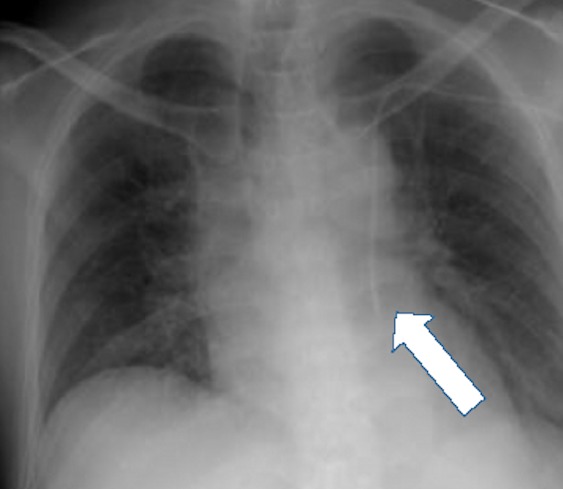
Portable chest radiograph showing a central line inserted in the left subclavian vein, catheter located at persistent left-sided superior vena cava (arrow). Patient was unaware of his congenital variant. He was always asymptomatic during line placement.

Other experts attribute malpositioning to variations in the venous anatomy. These variations can lead to catheter misguidance into vein tributaries that offer low-resistance routes for the entering catheter tip. Two types of variants in venous anatomy are recognized: congenital and acquired. In patients with CVCs, congenital variations are usually discovered incidentally on imaging after CVC placement.^11^ Although these variations are usually asymptomatic, they can make the radiologic location of the CVC tip difficult to discern. A common congenital variation with clinical significance is a persistent left-sided SVC (Figure 1 and 2), which is seen in 0.3% of healthy patients and 4.3% of patients with congenital heart disease.^12^,^13^ Other relevant congenital variations in venous anatomy include a dominant supreme (highest) intercostal venous drainage to the hemiazygos vein, dextrocardia, inferior vena cava variations, partial anomalous pulmonary venous drainage, and azygos vein abnormalities in origin, course, tributaries, anastomoses, and termination.^14^

**Figure 3:**
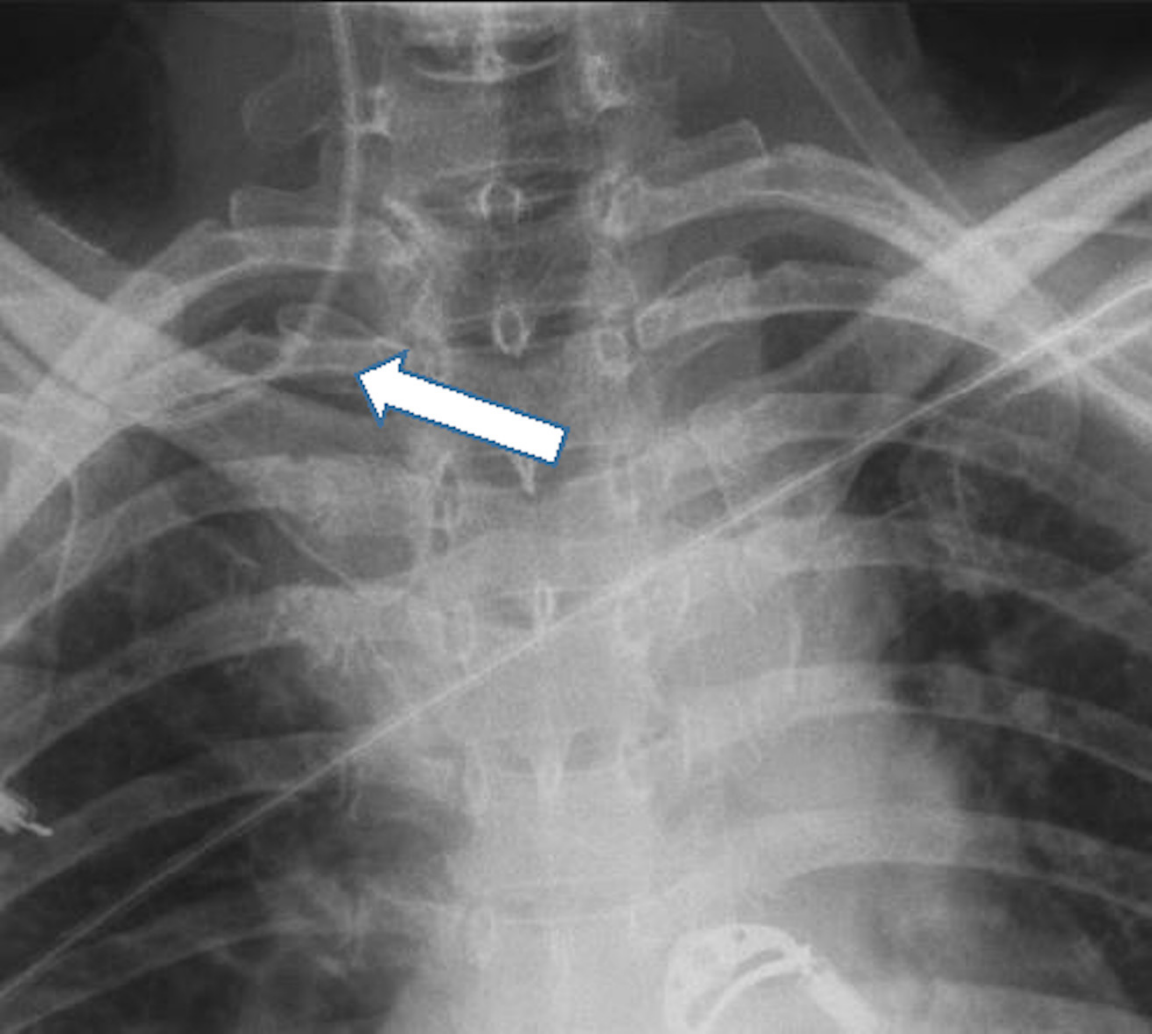
Portable chest radiograph showing a central line catheter placed on the right subclavian vein. The catheter migrated to the right internal jugular vein despite proper procedural technique. Mild resistance was experienced during the wire threading. The patient had history of chronic renal disease. A hemodialysis catheter had been placed in the right subclavian vein for several months and had been removed recently.

Acquired variations in venous anatomy are more common than congenital variations and can be external or internal in origin.^6^ More than 85% of external vessel distortions are caused by compression due to malignancy (often lung cancer, breast cancer, lymphoma, or germ cell tumors). Benign causes of external distortion include substernal goiter, thymoma, cystic hygroma, and histoplasmosis. Also, lung collapse or pleural effusions can shift venous structures such as the SVC away from the midline. Internal vessel distortion can be caused by thrombosis or stenosis. The risk of thrombosis can be increased by recent surgery, malignancy, immobilization, hemodialysis, chemotherapy, and pregnancy, and vessel stenosis has been associated with overuse of any vessel, subclavian cannulation, and central venous access from the left side of the neck.^15^ (Figure 3).

**Figure 4:**
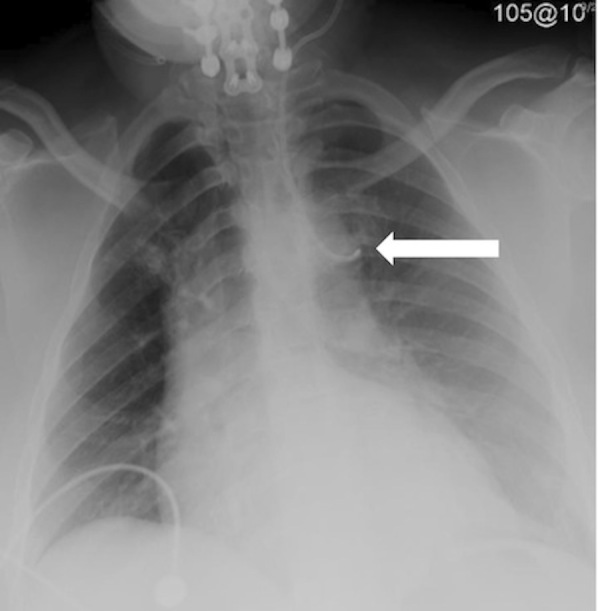
Portable chest radiography, limited by the patient’s body habitus, showing bilateral retrocardiac opacities and mild cardiomegaly. A left internal jugular central venous catheter extends through the hemiazygos vein; the catheter tip is most likely located in a left intercostal vein (arrow). A chart review revealed that the same malpositioning was present two months earlier. The patient experienced burning pain in the chest during a crystalloid bolus infusion.

CVC malpositioning is most common when a left internal jugular vein or subclavian vein is cannulated; a large prospective study by Schummer et al. of 1,794 central line catheterizations by experienced providers found that 6.7% of the catheter tips were intravenously malpositioned. Malposition was defined as CVC tip placement in a vein other than the SVC, or the right atrium, impingement with the lateral wall of the SVC (>40°) and arterial cannulation, most of which were inserted via the left internal jugular vein (12%), followed by right subclavian (9.3%), left subclavian (7.3%) and right internal jugular (4.3%).^3^ The increased risk of malpositioning with this approach is presumably due to the presence of a long left brachiocephalic vessel, a more oblique course to the heart, and the presence of small tributaries in that region (Figure 4).^16^ Using a CVC when its tip is located in a vessel of small diameter increases the risks of vascular perforation (incidence per catheter of 0.17%); other complications include catheter wedging, local venous thrombosis, catheter dysfunction, and cranial retrograde injection and should not be attempted.^4^

The probability of infection is another factor that has influenced the choice of CVC insertion site. It might explain why often a neck or thorax insertion site is preferred over a femoral one despite the presence of technical challenges. A randomized controlled trial comparing complications of femoral and subclavian venous catheterizations found that the femoral approach was associated with a higher incidence rate of infectious complications (19.8% vs. 4.5%; p<0.001); however, many lines were placed prior to the implementation of strategies for the reduction central line blood stream infections and the indications for catheter removal were not predetermined.^5^ In contrast, more recently, a meta-analysis by Marik et al. that included 113,652 catheter days showed no difference in the rates of catheter-related bloodstream infections between the femoral, subclavian, and internal jugular sites of cannulation.^17^ Timsit et al. reported similar results, the colonization was higher in the femoral lines; however, the infections were 1.0 per 1,000 catheter-days for the internal jugular site and 1.1 infections per 1,000 catheter-days for the femoral site, with a hazard ratio of 0.63 [0.25–1.63].^18^ None of these studies addressed CVCs inserted exclusively in the emergency department.

## PREVENTING MALPOSITIONING

### Selecting the vessel

The higher incidence of malpositioning in the left thoracic venous system than in the right side has been documented,^2^ which suggests that the right side of the circulation should be considered of first preference for CVC insertion unless those insertion sites are contraindicated. Ultrasound guidance can facilitate the identification of vessels but does not necessarily prevent CVC malpositioning.^19^ When anatomical distortion of vessels is known or suspected, the affected vessels should be avoided. The presence of scar tissue, thoracic tumors, or a history of recurrent cannulation or of long-term catheter placements (e.g., in patients on hemodialysis) should warrant caution (Figure 3).

### Choosing a technique

The appropriate catheter length should be selected on the basis of whether a right-sided or left-sided approach has been chosen. Improper catheter length increases the risk of catheter migration or displacement within the vessel.^20^ When a subclavian approach is used, making certain that the J-tip of the guide wire must be pointed caudad during insertion improves its successful guidance.^8^ Additionally, when a subclavian approach is attempted, lateral flexion of the head toward the insertion side narrows the os of the internal jugular vein, preventing the tip from entering the internal jugular circulation.^21^ Similarly, if the patient’s head is rotated away from the insertion site, then the internal jugular vein will be stretched and narrowed, which can maximize successful placement of the CVC in the intended vessel. Others have described the “finger in the fossa” technique of manually compressing the ipsilateral internal jugular vein to avoid its unintended cannulation.^22^

### Confirming placement

Proper CVC placement should be clinically verified, and also confirmed with diagnostic imaging. During cannulation of the internal jugular vein, a flush test may be useful for confirming adequate access. Flushing the CVC with 5–10mL of normal saline should result in a thrill felt on palpation or an audible bruit on auscultation at the internal jugular region, suggesting proper cannulation.^23^ Similarly, during a subclavian vein catheterization, the flush test has been used to accurately confirm correct tip placement; its presence in the neck could identify coiling in to the ipsilateral internal jugular circulation.^24^

Once a CVC is placed in the neck or thorax, radiography of the chest is the accepted way to confirm that the tip is adequately located in the atrio-caval junction and to rule out complications related to the procedure. Although, based on radiographic landmarks there is no clear consensus on the ideal positioning of the tip of the CVC, it is generally agreed that the tip should lie in the area of the junction of the SVC and right atrium to avoid contacting the pericardial reflection.^25^ This position is believed to minimize the risk of complications during clinical use. In general, the tip of a CVC should lie in the long axis of a wide vein with high blood flow, away from both the vessel wall and junctions.^26^

Furthermore, advances in ultrasonography have shown promising results for verifying CVC tip positioning. Various studies have shown that the ultrasonographic visualization of bubbles (seen as opacification) in the right atrium after injection of 10mL of agitated normal saline via the CVC port can be used to adequately verify placement of the CVC tip.^27^–^29^ Significant limitations of this technique include the inability to visualize the alignment of the catheter, and the presence of any aberrant course.

## IDENTIFIYING MALPOSITIONING THROUGH SIGNS AND SYMPTOMS

Inadequate catheter function and, less frequently, certain symptoms can indicate CVC malpositioning. Chest pain has been described in association with infusion through a malpositioned CVC in small tributaries of large central veins. For example, retrosternal pain radiated to the back with the infusion of hypertonic fluids in the left internal mammary vein has been documented in multiple case series.^30^ Pointing the tip of the catheter cephalad in the internal jugular vein and/or infusing near the intracranial structures can produce an “ear-gurgling” or “water running” sensation and headache,^31^ and infusing hypertonic solutions through a brachial vein can produce shoulder or arm pain.^30^

Another warning sign of malpositioning is insufficient blood return at entry ports owing to the collapse of weaker vein walls on the distal port when blood drawing creates negative pressure. However, the free return of venous blood does not guarantee proper CVC placement within a large vessel. Technical difficulty while threading the wire or inserting the catheter is an important sign of tip malpositioning. A lack of resistance to infusion is not a good indicator of malpositioning because positive pressure from the infusate easily overcomes the occlusion created by a malpositioned CVC. In a prospective observational study of patients undergoing CVC placement, Abood et al. found that clinical judgment based on comorbidities and the technical aspects of the procedure identified malpositioning correctly in only 20% of cases.^32^

## CONFIRMING MALPOSITIONING USING IMAGING

Anterior-posterior chest radiography after CVC placement is an important ancillary tool used to diagnose catheter malpositioning.^33^ Despite the high diagnostic accuracy of chest radiography for detecting CVC malpositioning, correct interpretation of these radiographs requires knowledge of the normal course and termination of mediastinal vessels related to the CVC.^34^

**Figure 5:**
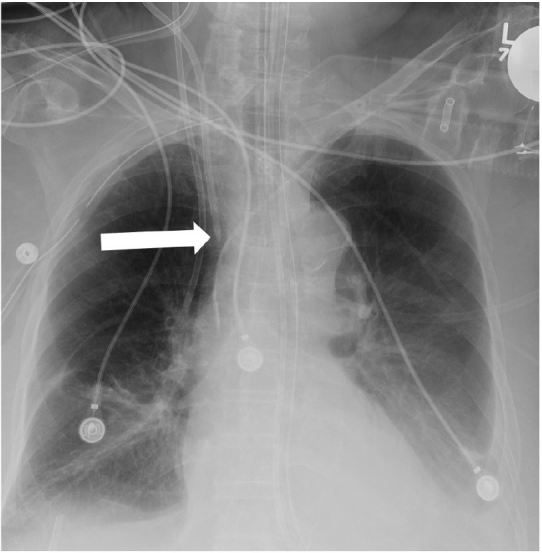
Portable chest radiograph showing mild cardiomegaly and bilateral basal lung opacities. A right internal jugular central venous catheter is shown with its tip apparently located at the superior vena cava. The emergency physician initially read the radiograph as showing the catheter as “adequately positioned.” Poor blood return was observed from the ports during placement. Chest burning pain was present during a normal saline infusion.

**Figure 6:**
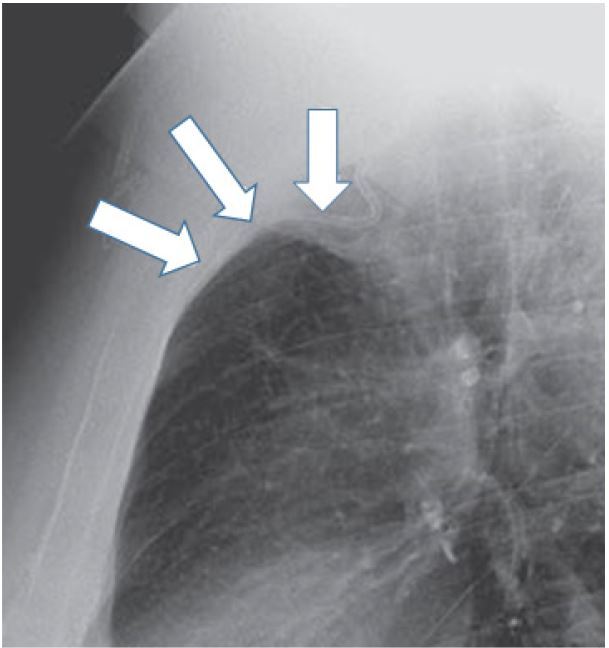
Lateral chest radiograph of patient in Figure 5 showing catheter (arrows) malposition coursing anteriorly along right internal mammary vein.

Undoubtedly, the 2D projections produced by conventional radiography, in contrast to those of computed tomography (CT), have limitations; for instance, the anatomical proximity of vessels to other structures can obscure whether the distal section of the catheter is in the intended location. If the CVC placement appears atypical on an anterior-posterior chest radiograph, then a lateral radiograph may be helpful. If there is still uncertainty, injecting a small amount of contrast material through the catheter during conventional radiography or performing CT may be necessary for precise radiographic localization.^35^ For example, when a right internal jugular approach is used, the tip of the catheter may occasionally be malpositioned in the internal mammary vein. Anatomically, the internal mammary vein originates from the brachiocephalic vein, which overlies the SVC and travels along the posterior aspect of the anterior chest wall.^36^ Thus, a catheter tip in the right internal mammary vein may appear to be within the SVC on a standard anterior-posterior chest radiograph (Figure 5, 6). CT, although expensive and impractical for routine use, can provide more definitive information than conventional radiography and is very useful for guiding the management of complications of CVC placement.

Another imaging technique used to diagnose CVC malpositioning is real-time radiograph imaging, which uses an image intensifier. This technique can guide wires and catheters centrally during CVC placement without the injection of contrast; unfortunately, its limitations are similar to those of plain radiography.^37^

## FIXING MALPOSITIONING

The consensus among experts is that a malpositioned CVC is suboptimal. In most circumstances, if a catheter is malpositioned, a priority should be to reposition, replace, or remove as soon as it is practical.^26^,^38^ In patients with difficult venous access and/or a high-value catheter, an objective evaluation of the risk-benefit situation should be done in order to determine whether to use the placed CVC. In addition, the insertion of a new catheter can be attempted after partial retrieval and redirecting of the wire guide, which may correct the malpositioning. Interestingly, in a prospective study in infants performed by Rastogi et al., in which 187 catheters inserted for long-term use were placed with a success rate of 98.9%, seven of all catheter tips were initially malpositioned (three in each internal jugular vein and one in the brachiocephalic vein), but all seven corrected themselves within one day, and a peripheral intravenous line was used until then in each case. The authors suggested that to avoid the stress of removing and replacing a malpositioned CVC, the CVC should be left in place since spontaneous correction may occur within one day.^39^ No similar observation has been described in adults. For catheters cannulated in noncompressible arteries or those centrally visualized but not clearly intravascularly located, it is prudent to seek guidance from interventional radiology or vascular surgery specialists before removing the CVC. The infusion of hyperosmolar solutions or vasopressors through the CVC increases complications associated with a malpositioned CVC and should be avoided before the malpositioning is fixed. Specific complications of a neglected malpositioned CVC should be managed on an individual basis, depending on the patient and presentation.

## CONCLUSION

CVC malpositioning is affected by congenital and acquired anatomical variants and by the techniques used to place and confirm placement of the CVC. Knowing the mechanisms of CVC malpositioning and how to prevent, identify, and correct CVC malpositioning could decrease harm to patients with this condition. Signs and symptoms of CVC malpositioning in a patient with apparently adequate CVC placement on plain radiographs should prompt more advanced diagnostic techniques. In general, repositioning, replacing or removing a malpositioned CVC should be done as soon as is possible.
